# 19-Year-Old with Sudden Onset Left Testicular Pain

**DOI:** 10.5811/cpcem.2022.7.56747

**Published:** 2022-10-24

**Authors:** Elan Small, Nicholas Ashenburg, Kimberly Schertzer

**Affiliations:** Stanford University School of Medicine, Department of Emergency Medicine Residency, Palo Alto, California

**Keywords:** point-of-care ultrasound, testicular torsion, testicle pain

## Abstract

**Case Presentation:**

A previously healthy 19-year-old man presented to the emergency department with severe, sudden onset of left testicular pain. Physical exam revealed a left high-riding, horizontally oriented testicle without cremasteric reflex. Point-of-care ultrasound was used to confirm the diagnosis of testicular torsion, as well as to guide manual detorsion, verifying return of blood flow after reduction.

**Discussion:**

Testicular torsion is a urologic emergency in which testicular viability is time dependent. Point-of-care ultrasound can be an important and helpful tool to not only confirm suspicion but help guide adequacy of blood flow return after manual detorsion in conjunction with comprehensive ultrasound.

## CASE PRESENTATION

**Image 1:**
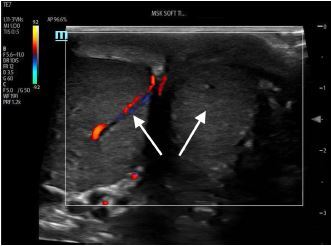
Side-by-side view of initial testicular assessment using point-of-care ultrasound. The testicle on the image-right corresponds to the patient’s painful left testicle which was found to be enlarged with surrounding edema and absent Doppler flow. Arrows highlight presence of flow in unaffected testicle and absence of flow in affected testicle.

**Image 2:**
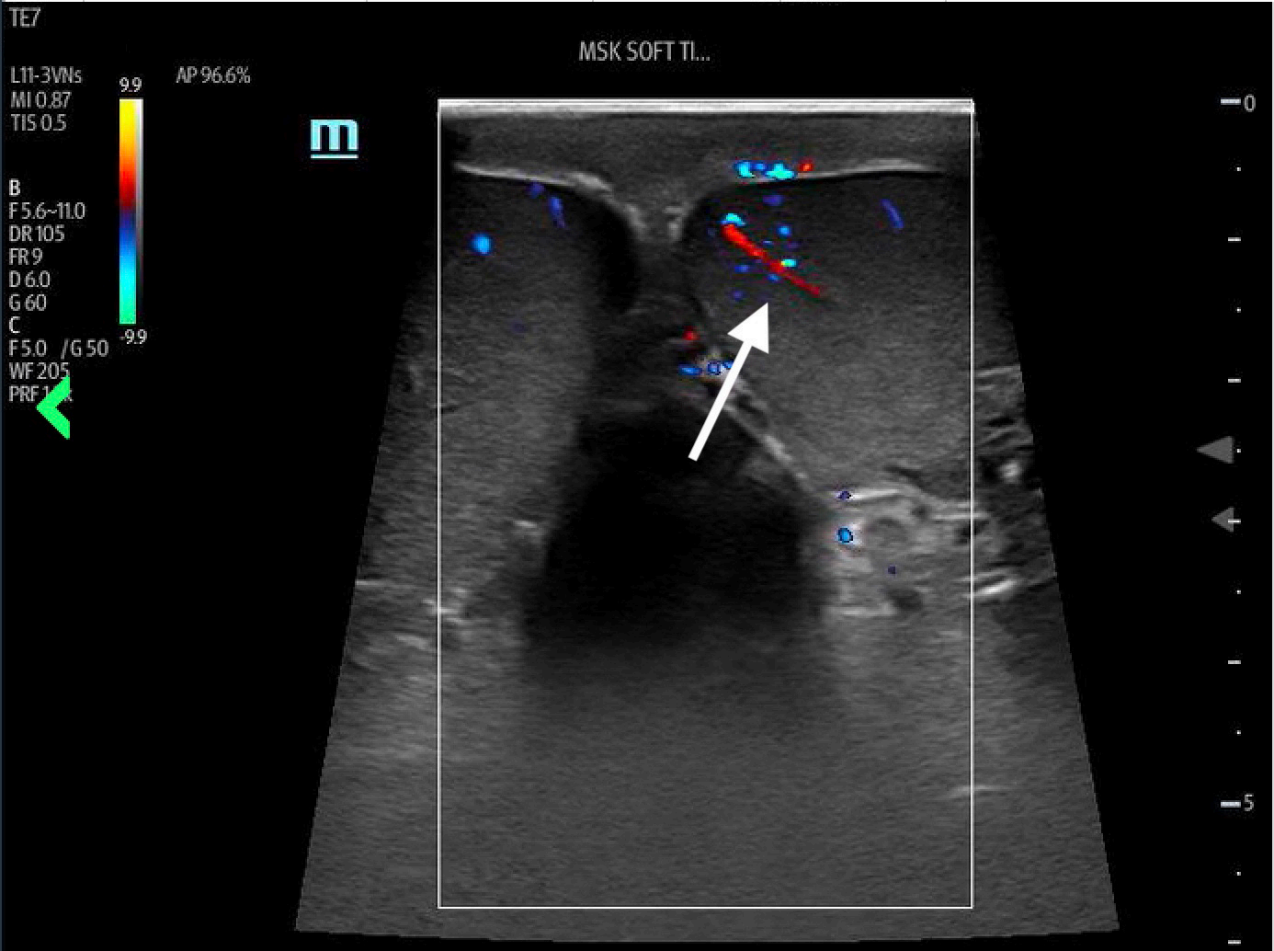
Side-by-side view of testicular assessment with point-of-care ultrasound after manual detorsion. The testicle on the image-right corresponds to the patient’s left testicle, with bilateral appreciable Doppler flow highlighted by the included arrow.

**Image 3:**
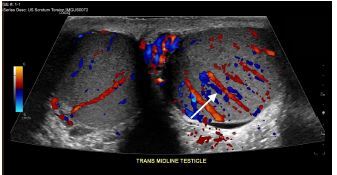
Comprehensive ultrasonographic side-by-side view of testicles after manual detorsion. The image-right corresponds to the patient’s left testicle, which demonstrated hyperemia likely related to detorsion event highlighted by the included arrow. Surrounding edema over affected testicle is also seen.

A 19-year-old man was brought by ambulance to the emergency department with left-sided testicular pain. He reported sudden severe, non-radiating left testicular pain that woke him from sleep 30 minutes prior to arrival. He presented in severe pain, tremulous from discomfort. His exam revealed a high-riding, firm, left testicle in a horizontal lie with absent cremasteric reflex. Urology was consulted and a comprehensive ultrasound ordered. The patient’s testicles were immediately examined with point-of-care ultrasound (POCUS) (Image 1). After receiving 100 micrograms of fentanyl, manual detorsion was conducted, and repeat ultrasonography was performed (Image 2). The patient was then transported for comprehensive ultrasonography (Image 3).

## DISCUSSION

Testicular torsion is a urologic emergency in which the testicle rotates 180–720 degrees, compromising venous and ultimately arterial circulation, leading to ischemia, necrosis, and nonviability. The literature has demonstrated good survivability at six hours, although it is time dependent with improved outcomes at quicker intervention.^1^ Classic history and exam features include sudden onset of pain, and a firm, horizontally oriented testicle with absent cremasteric reflex.^2^ Conventional teaching invokes early urologic consultation in highly suspicious cases prior to comprehensive ultrasonography.

POCUS has emerged as an important diagnostic tool, with up to 100% sensitivity reported in small-sample studies for both fellowship- and non-fellowship trained emergency physicians.^3^,^4^ Ultrasonographic features can include the following: an enlarged edematous hypoechoic testicle without Doppler flow; a whirlpool sign (spiral twist of the spermatic cord at the external inguinal ring or scrotal sac); and epidydimal enlargement without hyperemia.^5^ This patient underwent bedside manual detorsion with return of flow after approximately two lateral rotations. Comprehensive ultrasound demonstrated a hyperemic testicle consistent with recent detorsion. Depending on availability of resources and timing, POCUS can be an important tool in identifying testicular torsion and helping guide adequacy of reduction in conjunction with comprehensive ultrasonography.

**CPC-EM Capsule**

What do we already know about this clinical entity?

*Testicular torsion is a time-sensitive emergent condition for which point-of-care ultrasound has emerged as a potential tool for both identification and management*.

What is the major impact of the image(s)?

*These images illustrate classic ultrasound findings of testicle edema, and absence of Doppler flow and demonstrate return of Doppler flow after attempted detorsion*.

How might this improve emergency medicine practice?

*Depending on resources, providers should consider point-of-care ultrasound, paired with comprehensive studies, to identify testicular torsion and confirm detorsion*.
